# Evidence for long-range spatiotemporal interactions in infant and adult visual cortex

**DOI:** 10.1167/17.6.12

**Published:** 2017-06-16

**Authors:** Anthony M. Norcia, Francesca Pei, Peter J. Kohler

**Affiliations:** Department of Psychology, Stanford University, Stanford, CA, USA; Department of Psychology, Stanford University, Stanford, CA, USA; Department of Psychiatry, Stanford University, Stanford, CA, USA; Department of Psychology, Stanford University, Stanford, CA, USA

**Keywords:** *spatiotemporal interaction*, *apparent motion*, *development*, *visual evoked potentials*, *attention*, *human*

## Abstract

The development of spatiotemporal interactions giving rise to classical receptive field properties has been well studied in animal models, but little is known about the development of putative nonclassical mechanisms in any species. Here we used visual evoked potentials to study the developmental status of spatiotemporal interactions for stimuli that were biased to engage long-range spatiotemporal integration mechanisms. We compared responses to widely spaced stimuli presented either in temporal succession or at the same time. The former configuration elicits a percept of apparent motion in adults but the latter does not. Component flash responses were summed to make a linear prediction (no spatiotemporal interaction) for comparison with the measured evoked responses to sequential or simultaneous flash conditions. In adults, linear summation of the separate flash responses measured with 40% contrast stimuli predicted sequential flash responses twice as large as those measured, indicating that the response measured under apparent motion conditions is subadditive. Simultaneous-flash responses at the same spatial separation were also subadditive, but substantially less so. The subadditivity in both cases could be modeled as a simple multiplicative gain term across all electrodes and time points. In infants aged 3–8 months, responses to the stimuli used in adults were similar to their linear predictions at 40%, but the responses measured at 80% contrast resembled the subadditive responses of the adults for both sequential and simultaneous flash conditions. We interpret the developmental data as indicating that adult-like long-range spatiotemporal interactions can be demonstrated by 3–8 months, once stimulus contrast is high enough.

## Introduction

Spatiotemporal variations in the retinal image under natural viewing conditions come about from a combination of observer motion, eye movements, and importantly, from the movement of objects in the environment. In the case of a stationary, fixating observer, variations are caused predominantly by motion of objects in the visual environment. A central concern of the motion processing literature has been the number and kind of motion processing subsystems. An early distinction was made between short-range and long-range motion mechanisms (Anstis, 1980; Braddick, 1974; Cavanagh & Mather, 1989), but see (Cavanagh, 1991). The short-range system was posited to be comprised of a dense array of localized filters that performed an energy-like computation (Adelson & Bergen, 1985; Reichardt, 1957; van Santen & Sperling, 1985; Watson & Ahumada, 1985) to extract local directional signals. This system was contrasted with a second system that could operate on widely separated image tokens with very different properties, such as token shape or orientation, defeating computation in small, localized energy filters. In both the short- and long-range motion literatures, stimuli undergoing apparent, rather than real, motion have been used extensively because apparent motion paradigms allow for the parametric manipulation of spatial and temporal separation.

While there is ample evidence from single-unit recordings in both cat and monkey for energy-based motion computations in early visual cortex (An et al., 2012; Emerson, Bergen, & Adelson, 1992; Pack, Conway, Born, & Livingstone, 2006), single-unit correlates of long-range apparent motion have not been found. However, other work in cat (Jancke, Chavane, Naaman, & Grinvald, 2004) and ferret (Ahmed et al., 2008) using voltage-sensitive dye imaging (VSDI) has suggested a possible substrate for long-range motion may exist in a second, qualitatively different type of direction selectivity that is represented in the spatiotemporal pattern of activity in large populations. In experiments with long-range apparent motion sequences (Ahmed et al., 2008), a travelling wave of activity in the direction that humans perceived motion was observed, propagated along the cortical surface from the retinotopic projection of the first stimulus patch in a motion sequence to the location of the second patch.

Developmentally, there is ample evidence for the presence of direction-selective neurons that compute motion direction based on local motion energy. In macaque, direction selectivity can be found as early as 6 days (Chino, Smith, Hatta, & Cheng, 1997). In human, visual evoked potential (VEP) correlates of motion selectivity have been found as early as 6 weeks (Birch, Fawcett, & Stager, 2000) based on the monocular nasalward/temporalward motion asymmetry (Norcia et al., 1991) or at about 9 weeks of age based on direction-shift responses (Wattam-Bell, 1991). The case of long-range motion is interesting from a developmental perspective in that it may involve mechanisms different from motion energy filters in early visual cortex that are established near or soon after birth, such as those implicated in the VSDI studies in cat and ferret. Moreover, fMRI studies in humans have found a range of extrastriate areas are involved in the percept of long-range apparent motion (Larsen, Kyllingsbaek, Law, & Bundesen, 2005; Larsen, Madsen, Lund, & Bundesen, 2006; Muckli, Kohler, Kriegeskorte, & Singer, 2005; Muckli et al., 2002; Sterzer & Kleinschmidt, 2007; Sterzer, Russ, Preibisch, & Kleinschmidt, 2002; Zhuo et al., 2003). Taken together, these results suggest that larger-scale integrative activity may underlie the processing of long-range motion and given this, it is possible that these mechanisms might not be functional in early infancy due to previously reported immaturities in extrastriate cortical areas such as those reported for the macaque (Batardiere et al., 2002; Rodman, 1994).

To probe for the existence of putative long-range motion mechanisms during infancy, in this study we used stimuli that, while being a poor match to the properties of classical direction-selective receptive field mechanisms in early visual cortex, nonetheless give rise to a robust percept of apparent motion in adults. The direction of motion in our stimuli was parallel to the orientation axis of small-patch stimuli, rather than being orthogonal to it. This disrupts the typical relationship between direction of motion and the orientation tuning of direction selective cells in macaque (Albright, 1984). We used a VEP paradigm similar to one used in the VSDI recordings in order to make comparisons between scalp-recorded activity and the activity in early visual cortex recorded under similar stimulation conditions. The goal of the experiments was to probe for the presence of spatiotemporal interactions of sufficient spatial range to possibly mediate long-range motion percepts in both adults and in infants. We do not explicitly link neural activity to perception of motion here, as that was not possible to do in infants. Rather, we ask whether similar or different patterns of spatiotemporal interaction are present in both infants and adults under conditions that either give rise to or do not give rise to a percept of motion in adults.

## Methods

### Participants

A total of 27 neurotypical adults (ages 18 to 57, *M* = 26.9 years) participated. Data from 12 participants (six women, six men) collected from a subset of conditions in the main experiment were used to constrain the electrodes and time-points of interest for the analysis of the main experiment, which was conducted in 14 new participants (five women, nine men). Each participant had best-corrected distance visual acuity of 6/6 or better on the Bailey-Lovie constant LogMAR chart and normal stereo-acuity on the RandDot stereotest. A total of 50 typically developing infants participated, with 43 producing usable data. Twenty-six participated in the lower contrast version of the experiment, with 23 infants producing usable data (*M* = 5.06 months, range 3.5–6.5 months, ±0.72 *SD*; nine girls, 14 boys). Twenty-four infants participated in the higher contrast version, with 20 producing usable data (*M* = 5.5 months, range 4.0–8.4 months, ±1.62 *SD*; 10 girls, 10 boys). Each adult participant or the parent of the infants gave informed consent after having the procedures of the study explained using a consent form and protocol approved by the Institutional Review Board of Stanford University.

### Visual display

**Figure 1:**
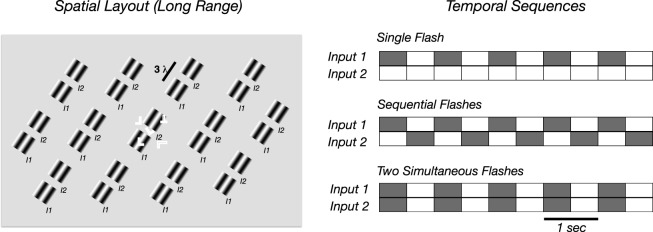
Schematic illustration of long-range stimuli used in the experiments. (Left panel) Spatial layout of patches used in the long-range sequential flash and long range simultaneous flash conditions where the patches were separated by 3λ along the orientation axis. In adults, a letter array was superimposed and was present under all conditions. The letters were task-relevant in a subset of the conditions. (Right panel) Temporal sequences for single flash, sequential flash, and two simultaneous flash conditions. The temporal period of the display was 1 s and trials lasted 10 s. Inputs 1 and 2 are the separate sets of 13 patches comprising the complete display. In the single input case, only one set of 13 patches was presented. In the apparent motion sequences, the 13 patches were presented sequentially and in the two flash conditions, the 26 patches were presented simultaneously.

The stimuli consisted of arrays of windowed, 2 c/° sine-wave gratings (1° × 1°) arranged on a hexagonal lattice. The stimuli were presented on a gamma-corrected CRT monitor (HP p1230; Hewlett Packard, Palo Alto, CA) at a screen resolution of 800 × 600 pixels and a monitor refresh rate of 72 Hz. The patches appeared from an equiluminant gray background of 58 cd/m^2^ at a temporal frequency of 1 Hz and a Michelson contrast of 40% in the main experiments. The stimulus array is shown schematically in Figure 1. The patches were centered on a hexagonal grid of 6.0° lattice spacing. The horizontal and vertical extent of the grating-patch array was 16° × 12° at a viewing distance of 127 cm.

In the main experimental conditions, two grating patch arrays were presented with a spatial offset of the patch centers of 1.5° (3 wavelengths of the 2 c/° carrier grating). The orientation of the grating inside each patch was parallel to the line connecting the centers of the nearest neighboring patches (e.g., the patches were coaxial and the carriers were collinear). In the sequential flash sequences, the patches were presented at the first location for 500 ms and then, immediately after their offset, they were presented at the second location. This condition led to a percept of apparent motion in adults. In control conditions, each group of patches was presented independently at each of the two spatial offsets in the adults and in a subset of 12 of the infants. The remainder of the infants were presented with a single patch array at one location. Responses generated by sequential flash/apparent motion sequences were compared to those in another condition in which the two sets of patches appeared simultaneously (simultaneous flash condition). In a second sequential flash condition shown to adults (short-range condition), the patches were presented at a spatial separation of 0.125° (e.g., 90° of spatial phase, or 0.25 wavelength of the carrier grating). The spatial offset of the two patch arrays in the short-range condition was in a direction orthogonal to the orientation axis of the carrier grating. Thus, the motion direction in this condition was consistent with the typical correlation between orientation and direction selectivity in early visual cortex (Albright, 1984). The short-range stimulus design thus favored the activation of motion energy processes. Finally, the two sets of patches were spatially superimposed and presented at the same time (simultaneous short-range flashes).

### Procedure for adults

The adult participants viewed the display monitor binocularly from a viewing distance of 127 cm. Attention was varied through two different task instructions. In the attend letters task, designed to divert attention away from the VEP stimuli, the participants performed a difficult letter discrimination task placed over the central 1.3° × 1.3° of the visual field. Target letters were presented at five locations, four at the corners of a square with the fifth letter presented in the middle of the square (see Figure 1 for a schematic illustration). The target arrays were preceded and followed by matching arrays comprised of letter F characters designed to signal the upcoming onset of the array and to mask the target array to control its visibility via a target-array duration parameter. The target array either consisted of five L characters, or four L and one T character. The two target-array types occurred equally often and in random order. The orientation of each of the letters was random and independent at each of the five locations. To control task difficulty at a constant level, the exposure duration of the target array was placed on a staircase that was designed to hold performance at 82% correct. The participants indicated their choice (T present or absent) with separate button presses. In the attend flashes conditions, the observers were instructed to fixate the central letter of the letter array, but to spread their attention to the flashing patterns. Single trials consisted of 12 s presentations of the patches. The initial and final 1 s of the trial presentation was discarded from the analysis, and cycle-averages (1000 ms) were constructed over 10 trials in each stimulus condition (100 s of data per condition per observer).

### Procedure for infants

Infants were seated comfortably on their parent's lap at 127 cm from the screen. The trial durations were the same as for adults. The infant's attention was attracted to the center of the screen with a small noisy fixation toy. Fixation on the center of the screen was determined by an experimenter located behind the screen who could monitor the centration of the image of the video monitor in the infant's pupil. Trials were interrupted when fixation was lost and resumed when it was regained. In one version of the experiment, run at 40% contrast, two recording sessions were performed with either five or six experimental conditions and five trials per condition (10 trials per condition were thus recorded over the two sessions). The five-condition experiment (*N* = 11) comprised one single-patch condition, long-range and short-range sequential patch conditions, and long- and short-range simultaneous patch conditions. The six-condition experiment (*N* = 12) added a second single-patch condition. The second version of the experiment used 80% contrast stimuli and was run with the single-patch condition and a long-range motion and long-range flash condition, each with 10 trials collected in a single session. In cases in which the data were collected in two sessions, the data were recorded within two weeks of each other. An infant's data were included if at least five trials per condition were successfully recorded. This was not possible in seven out of 50 infants. Of the 43 infants included in the data analysis, 38 had the full 10 trials per condition and five infants had five trials per condition.

### EEG acquisition and processing

The electroencephalogram (EEG) was recorded over 128 channels using HydroCel electrode arrays and an Electrical Geodesics NetAmp300 (Electrical Geodesics, Eugene, OR). The sampling rate was 420 Hz. Triggers indicating the onset of the display sequence were registered as digital inputs with 1 ms precision. The data were recorded over a 0.3–50.0 Hz passband. Artifact rejection was performed in two steps. First, the continuous filtered data were evaluated by a sample-by-sample thresholding procedure to locate consistently noisy sensors. These were replaced by the average of their six nearest spatial neighbors. Second, once noisy electrodes were substituted, the EEG was re-referenced from the Cz reference used during the recording to the common average of all the sensors. Finally, EEG epochs that contained a large percentage of data samples exceeding threshold (∼30–80 microvolts for adults, 200 microvolts for infants) were excluded on a sensor-by-sensor basis. Trial data were signal averaged over a 1-s cycle for visualization purposes and over a 2-s epoch containing two cycles of the evoked response for spectral filtering and reconstruction of the cycle-averaged time-course via inverse Fourier transformation.

The response spectra in the apparent motion conditions were strongly dominated by activity at even harmonics of the 1 Hz stimulation rate, consistent with the symmetry of the apparent motion percept. To increase the signal-to-noise ratio of the records and to minimize components of noninterest, such as small amplitude differences between responses to different sets of stimulus patches, time averages were reconstructed by back-transforming only the even harmonics of the response up to 52 Hz (one eighth of the sampling frequency). Responses for the simultaneous two-flash conditions were reconstructed using all harmonics up to 53 Hz because the pattern onset/offset VEP is dominated by the onset response and thus contains strong odd and even harmonic components.

To reduce the dimensionality of the 128 channel scalp data in a principled way, we derived a set of five electrode regions of interest (ROIs) from independent data. These ROIs were based on an atlas of scalp topographies generated from a group of 20 participants who underwent fMRI mapping of a set of retinotopic (V1, V2, V3, V3A, and V4) and functionally defined (hMT+ and lateral occipital) visual areas. For each visual area, an elementary, current dipole of unit amplitude was placed at each 3D vertex in the cortical surface for each participant. The lead-field matrix for each individual was used to create a map of the expected electrical potential on the sensor array for that visual area. These maps were then averaged across individuals to create a group topography for each visual area (for details of the procedure, see Ales, Yates, & Norcia, 2010). The five-electrode ROIs were selected as being representative of activity from early visual cortex (occipital pole [OP]; EGI sensor 75/Oz), area V3A (dorsal occipital [DO]; EGI sensors 60, 67, 77, 85), and area hMT+ (temporal occipital [TO], EGI sensors 51, 97). The hMT+ ROI was derived from a moving versus stationary, low contrast random dot motion localizer (Appelbaum, Wade, Vildavski, Pettet, & Norcia, 2006). A lateral occipital ROI (LO; EGI sensors 58, 65, 90, 96) was derived from an intact versus scrambled object fMRI localizer (Appelbaum et al., 2006; Kourtzi & Kanwisher, 2000). Because previous work with higher-order motion stimuli has shown parietal activations (Claeys, Lindsey, De Schutter, & Orban, 2003; Orban et al., 2003), a posterior parietal cortex ROI located anterior to the DO ROI (PAR; EGI sensors 31, 37, 53, 54, 55, 60, 61, 62, 67, 72, 77, 78, 79, 80, 85, 86, 87) was defined.

### Model testing

The nature of spatiotemporal interaction underlying the evoked responses was probed by forming linear predictions based on single patch responses. Linear predictions for the sequential flash responses were formed by first back-transforming the even harmonic components (up to 52 Hz) of the single-flash responses and then adding them with a 180° phase shift to account for the stimulus sequence. Linear predictions for the simultaneous flash conditions were formed by simple addition of the back-transformed single-flash responses reconstructed over the integer harmonics between 1 and 53 Hz. The ability of the linear predictions or scaled versions of them to match the measured data was assessed via calculating difference potentials between the predicted and measured data. Deviations from the prediction were assessed via run-corrected *t* tests. Permutation methods (Blair & Karniski, 1993) were used to form an empirical sampling distribution for the difference, which was tested against zero after correcting for runs. For every permutation, we computed point-by-point *t* scores for the waveform difference, and found the longest run of consecutive time points with *p* values less than 0.05. This procedure generates a nonparametric reference distribution of consecutive significant *p* values. A run-corrected significant difference at the *p* < 0.05 level was declared if the length of any consecutive sequence of significant *t* scores in the original, nonpermuted data exceeded 95% of the values in the null distribution. This test also localizes the time periods when such significant differences occur. However, since choice of keeping family-wise error at 5% is arbitrary and conservative, we also present the uncorrected significance values (see red/yellow color maps in Figures 3, 4, 6, 7, and 8).

## Results

Our analysis of spatiotemporal interactions in adults and infants begins with a comparison of their responses to single flashes and then proceeds to an analysis of measured responses to sequential and simultaneous paired flashes, each compared to a corresponding linear prediction formed from the single flash responses. Here and in the following sections we will use the following terminology to refer to the stimulus conditions. The term *single flash* refers to the presentation of one set of 13 grating patches, *sequential flashes* refers to the case in which the two single flashes were presented in temporal alternation, and *simultaneous flashes* refers to the case in which both sets of patches were presented at the same time rather than in temporal alternation.

### Single-flash responses in infants and adults

**Figure 2:**
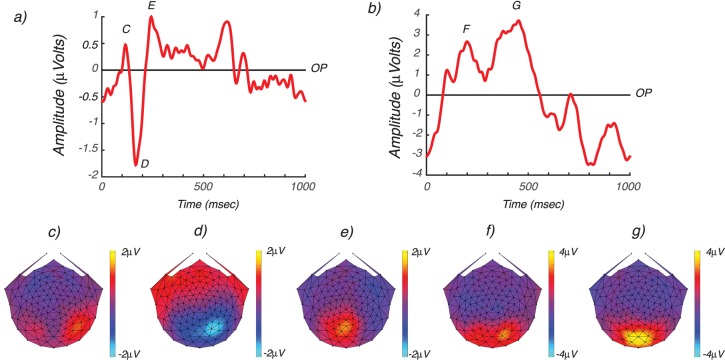
Comparison of adult and infant single flash response waveforms and topography. (a) Adult VEP waveform for OP electrodes. The response consists of multiple response peaks (P100, N170, P250) as indicted by upper case C, D, and E. (b) Infant VEP waveform for OP electrodes. The response is dominated by two positive peaks at 150 (F) and 240 (G) ms. (c–g) Scalp topographies corresponding to the response peaks in (a) and (b).

Figure 2a shows adult single flash responses for electrodes over the occipital pole (OP). Comparable data for infants are shown in Figure 2b. These responses consist of pattern-onset, transient VEPs. The adult pattern onset response is comprised of a positivity near 100 ms that has a lateralized scalp topography (Figure 2c). This positivity is followed by a larger negativity, peaking around 170 ms that is right-lateralized (Figure 2d). This negativity is followed by a second positivity peaking at around 250 ms that has a dorsomedial scalp topography (Figure 2e). These responses are broadly similar to other pattern onset VEPs measured in adults (Di Russo, Martinez, Sereno, Pitzalis, & Hillyard, 2002). By contrast, the infant responses are markedly different both in waveform and scalp distribution (see Figure 2b). Instead of a series of sharp, alternating polarity responses, the infant pattern-onset response comprised a broad positivity with positive subpeaks at approximately 150 ms and 420 ms. These scalp-positive responses were maximal on right lateral electrodes at 150 ms (Figure 2f) and at medial occipital electrodes at 420 ms (Figure 2g).

**Figure 3:**
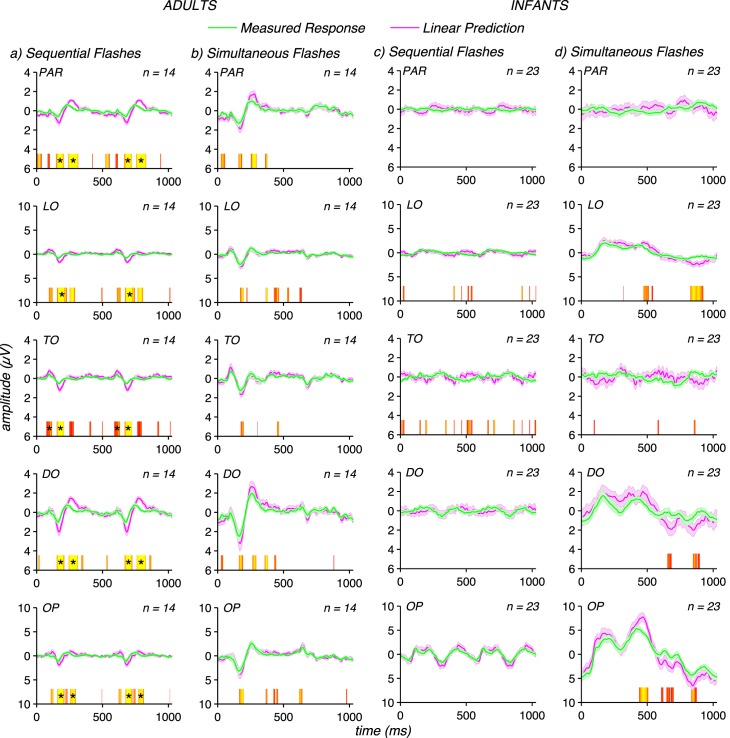
Comparisons of linear predictions to measured responses. (a) Linear predictions for adult sequential flash condition (magenta) are larger than measured responses (green) by a factor of ∼2. (b) Linear predictions for the adult responses to simultaneous flashes are a factor of ∼1.5 larger than the measured responses. (c) Linear predictions for infant sequential flash responses are approximately equal to the linear prediction. (d) Infant responses to simultaneous flashes are approximately equal to their linear prediction except at long latencies (∼450 ms). Waveforms are group means for each group (infants and adults) over five electrode ROIs (OP = occipital polar, DO = dorsal occipital, TO = temporal occipital, LO = lateral occipital, PAR = parietal), with error bars representing the standard error of the mean. Time points where the difference between measured and predicted responses reach significance at the *p* < 0.05 level are indicated above the abscissa by red to yellow color scale with yellow corresponding to lower, more significant *p*-values. Runs that remain significant at the *p* < 0.05 level after run correction are indicated with black stars.

Figure 3 plots measured response waveforms (green curves) for five electrode ROIs (OP, DO, TO, LO, and PAR) for both adults (left) and infants (right). In addition to the differences in response waveforms between infants and adults just described, the distribution of evoked response across ROIs also differs between infants and adults: infant responses are less widely distributed than those of the adults. These differences in topography may reflect the activity of a more restricted set of underlying sources in infants versus adults. However, current spread is expected to be more restricted in infants due to their higher skull conductivity and the smaller distance between electrodes and brain. In the absence of a detailed analysis of the infant head volume conductor, we cannot confidently discriminate these two alternatives and therefore we do not interpret these apparent differences further.

### Detecting spatiotemporal interactions in infants and adults

In Figure 3 we compare measured responses to sequential and simultaneous flashes to the corresponding linear predictions. The linear predictions were compared to the measured responses to detect the presence of nonlinear spatiotemporal interactions as deviations from the linear prediction. Deviations where the measured responses are smaller than the linear prediction are interpreted as being due to a suppressive interaction. A lack of deviation from the linear prediction was interpreted as a lack of spatiotemporal interaction. The linear predictions were formed by summing the single flash responses presented at each location. For the simultaneous flashes, the responses from the patches presented at each separate location were summed. For the sequential flashes, the responses were cyclically shifted by 180° to reflect the timing sequence of the sequential flashes. Infant responses to a single flash presented in one position were doubled in amplitude, as responses to both single flash locations were not measured in all participants.

In the sequential flash condition, the measured evoked responses in adults (Figure 3a, green curves) are smaller than the corresponding linear predictions (magenta curves). The sequential flashes gave rise to a percept of apparent motion in adults. Deviations from the linear prediction occur consistently during the negative-going peak at ∼170 ms (N170) where they are a factor of approximately two in amplitude at TO and DO, for example. Deviations are also present during the positive peak at ∼96 ms (P100) at TO and again during the second positive peak at ∼250 ms (P250) at DO. The measured evoked responses to simultaneous flashes (Figure 3b) are also subadditive in the adults, but less so than for sequential flashes. Simultaneous flashes do not appear to move. The most consistent point of deviation is during the N170 peak, but the differences there do not survive run correction.

By contrast to the responses of adults, the infant sequential and simultaneous flash responses are each well approximated by the corresponding linear predictions, with the only substantial deviation occurring for simultaneous flash responses at around 450 ms (Figure 3d), but that deviation does not survive run correction. In the simultaneous flash condition, the maximum deviation between measured and predicted responses was a factor of 1.3 at OP. We also formed the linear predictions from measurements of both isolated flash responses in a subset of infants (*N* = 12), as we did for adults, to verify that linear predictions made on the basis of only one of the sets of patches were valid. We found that the pattern of results seen for summing the responses from flashes presented at only one of the positions did not differ from those presented at both locations (data not shown). Note that we have not explicitly tested whether the size of the additivity failures differed between infants and adults. Making such a test is complicated by the large differences in response waveform and topography (see below) that make it difficult to extract a common set of response components over which the magnitudes of the additivity failures could be compared.

### Modeling the additivity failures

**Figure 4:**
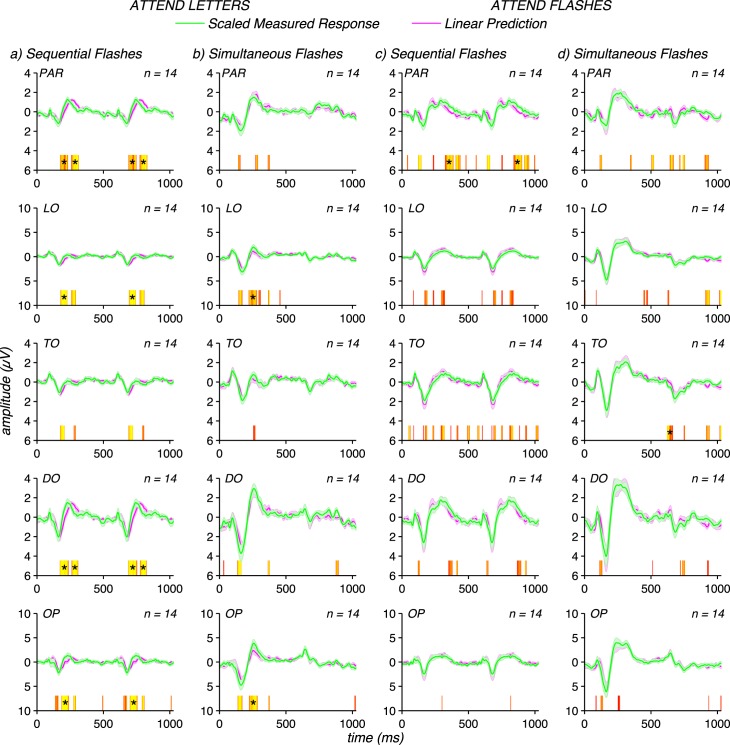
Scaled evoked responses match linear predictions reflecting the magnitude of subadditivity in the adult VEP. Panels (a) and (b) present data collected under the attend letters task instructions. Panels (c) and (d) present data collected under the attend flashes task instructions. Measured responses were scaled by a factor of 2 for the sequential flash conditions and by a factor of 1.5 for the simultaneous flash conditions. Measured single flash responses collected for each task instruction were used to form the corresponding linear predictions. Waveforms are group means for each adult data set over the five electrode ROIs described in the legend for Figure 3, with error bars representing the standard error of the mean. Significance of the differences between scaled responses and linear predictions are plotted above the abscissa, following the logic described for Figure 3.

**Figure 5:**
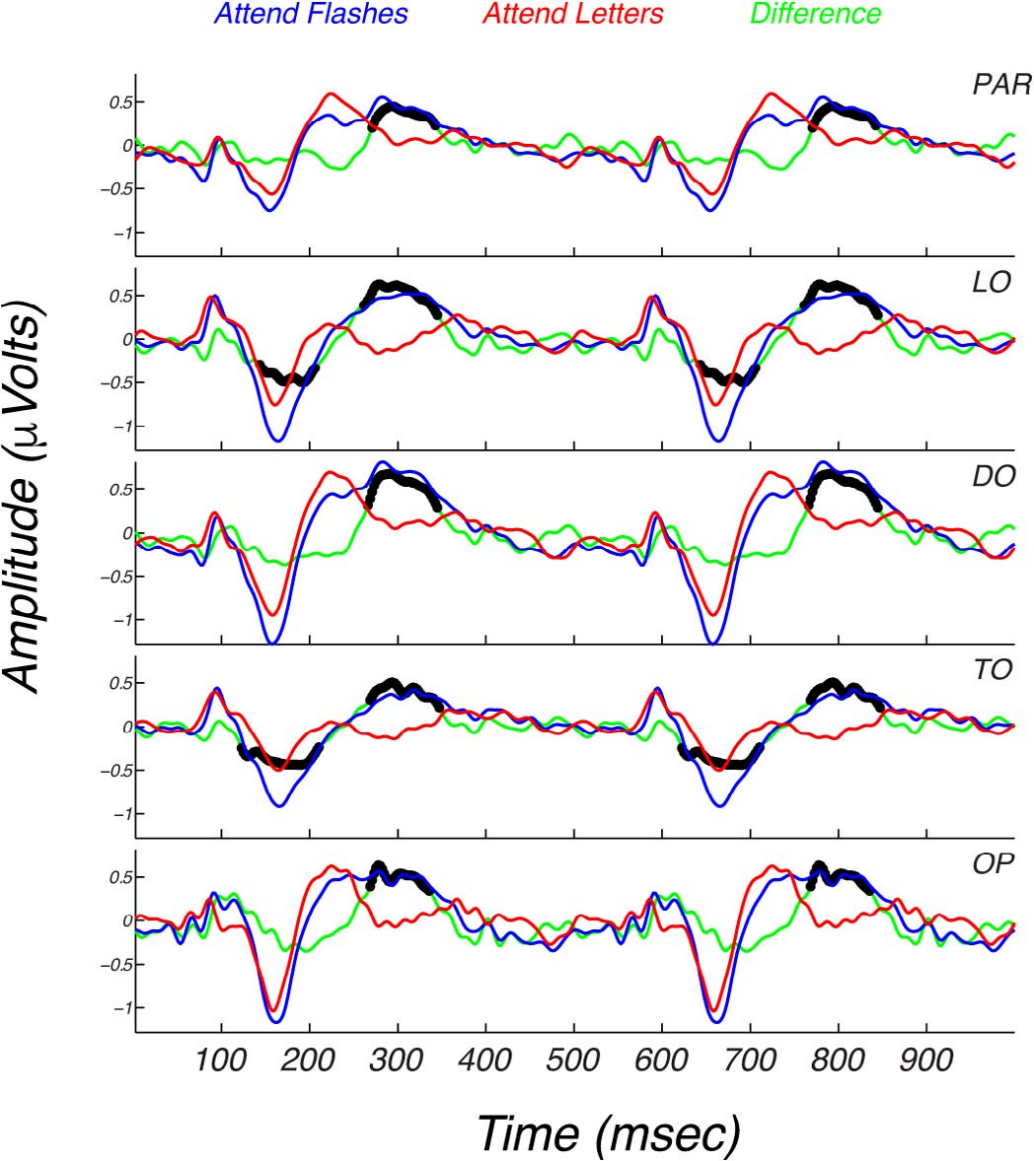
The effect of attention on sequential flash responses in five electrode ROIs. Group means of the adult data collected under the attend flashes task instruction are shown in blue, while red curves plot the same data for the attend letters task. The green curves plot the mean difference potentials. The black symbols on the curves reflect points of significant difference on run-corrected permutation tests (*p* < 0.05). The effect of attention is first seen at ∼125 ms at TO and LO sites. The effect of attention is largest at approximately 300 ms. Rows correspond to the five electrode ROIs described in the legend for Figure 3.

The suppressive nonlinearity in adults appears to be a multiplicative effect at all time points for both sequential and simultaneous flash conditions. To test this model, we first derived a single multiplicative scaling factor from an independent set of measurements in 12 observers for these two conditions, and applied the derived scale factors to the sequential flash data (a factor of 2) and simultaneous flash data (a factor of 1.5) for the main group of 14 observers. The same scaling factor was applied to each electrode ROI. This approach both sets the scale factors and tests the reproducibility of the effect as the factors are derived from independent data. The resulting comparisons are shown in Figure 4. For the sequential flashes in adults during the attend letters task (Figure 4a), the scaled data match the linear prediction for the P100 and N170 peaks. The scaling fails however on the trailing edge of the N170 peak. There is a delay of the P250 peak in the linear prediction relative to the measured response and this leads to a shallower slope from N170 to P250 in the predicted compared to the measured data. For the same condition under the attend flashes instruction, the only points that survive run correction are on the trailing edge of P250 at the PAR site. The effect of attention task will be evaluated directly in Figure 5.

The simultaneous flash data is well predicted by scaling the measured responses by a factor of 1.5 at all time points except for the P250 peak at OP and LO in the attend letters condition (Figure 4b). No deviations are present after run correction for the simultaneous flashes in the attend flashes condition (Figure 4d). Scaling the infant data by these factors would over-correct them, as their data are approximately linear without scaling (see Figure 3c and d). From this analysis, we conclude that spatiotemporal interaction is stronger for sequential compared to simultaneous flashes in adults, and that spatiotemporal interactions under both conditions are much weaker or unmeasurable in infants.

### The effect of attentional task in adults

An influential proposal in the motion processing literature is that motion mechanisms can be distinguished on grounds other than spatial separation alone. In this view, motion mechanisms are either *passive* or *active* (Cavanagh, 1991, 1992; Lu & Sperling, 1995, 2001; Verstraten, Cavanagh, & Labianca, 2000). The passive system is postulated to be a purely feed-forward process that utilizes a motion energy computation in a dense array of detectors to extract motion signals. By contrast, active mechanisms have been shown to be necessary in order to explain motion percepts that are present in specialized stimuli where energy computations do not produce a specific motion direction. The earliest demonstration of an active motion process came from Wertheimer (1912) using a stimulus consisting of two alternating crosses, one rotated by 45°. Wertheimer noticed the perceived direction of (ambiguous) motion could be altered at will. Interest in this perceptual phenomenon has enjoyed a renaissance in recent years (Cavanagh, 1992; Kohler, Haddad, Singer, & Muckli, 2008; Ramachandran & Anstis, 1983; Suzuki & Peterson, 2000; Verstraten et al., 2000).

In the recordings just described, the infants' attention was directed to the screen via a noisy fixation toy that may have divided their attention from the flashes and this could have reduced the magnitude of the observed spatiotemporal interaction. The difficult letter task was employed in the adults to mimic this situation and the data from adults presented in Figures 2, 3a, 3c, 4a, and 4b were collected under this set of task instructions. To assess the possible role of the availability of attentional resources, we also recorded evoked responses in the adults under conditions in which they were instructed to fixate and attend to the flashing stimuli under the assumption that the participants would be able to devote more attentional resources to the stimuli from which we were recording evoked responses.

Instructing adult participants to attend to the moving/flashing stimuli led to larger evoked responses as can be seen in Figure 5 which plots data from the five-electrode ROIs defined over the posterior scalp (OP, TO, DO, LO, and parietal). Data from the attend sequential flashes condition is plotted in blue; the attend letter condition in red; and the between-conditions difference in green. A measurable effect of attention first manifests over TO and LO electrodes along the downward slope from the positivity peaking at ∼100 ms to the negativity peaking at ∼160 ms (see green trace in Figure 5 that plots the difference potential). There are large negativities at the same latency at OP and DO electrodes, but there is no measurable difference attributable to attention at these sites or at the time of the initial positivity around 100 ms at any location. The effect of task is largest and most widespread during the interval of ∼280–320 ms. At this latency, a difference due to attentional task is present at all sites, including OP and DO sites and occurs during a major positivity.

To test whether the magnitude of the spatiotemporal interaction depends on attention, we repeated the scaling analysis for the letter task shown in Figures 4a and 4b, but this time for the attend sequential or attend simultaneous flash conditions. In this analysis, the single flash responses and thus, the linear predictions were derived from data collected under instructions to fixate and attend to the flashing stimuli. Here, a factor of two scaling of the measured sequential flash responses also equated them to their corresponding linear predictions (compare Figure 4a to Figure 4c) and a factor of 1.5 scaling of the simultaneous flash responses again equated them to their corresponding linear predictions (compare Figure 4b to Figure 4d). These results indicate that the patterns of subadditivity for sequential and simultaneous flash responses do not depend substantially on the nature of attentional deployment. The same factor of ∼2 subadditivity for sequential flashes occurs for both attentional tasks, as does the same factor of ∼1.5 subadditivity for simultaneous flashes.

### Effects of spatial separation and contrast

Infants could fail to show long-range spatiotemporal interactions for a number of reasons. Infants could simply lack the requisite anatomical connectivity/receptive field structure needed to integrate activity between the spatially separated patches. Alternatively, the nature of contrast coding could differ between infants and adults—infants could lack the mechanisms responsible for response subadditivity. Subadditivity, as measured in adults, could be due to the stimuli being processed through a saturating nonlinearity such as the sigmoidal nonlinearity common to normalization models of spatiotemporal interaction (Albrecht & Geisler, 1991; Heeger, 1992; Robson, 1988). Finally, infant contrast sensitivity measured with the VEP is known to be immature at 4–8 months of age (Norcia, Tyler, & Hamer, 1990) and this may place the stimuli on a lower, nonsaturated portion of the contrast response function.

As one check on a possible role of immature contrast processing, we included two “short-range” conditions, one in which the patches were separated of 0.25 wavelength in a direction orthogonal to the orientation axis and the second where the patches were presented with no spatial separation (doubling the contrast of the patches). The first stimulus configuration places both flashes within the classical receptive fields of putative motion energy detectors and the spatial separation is optimal for motion energy units (Nakayama & Silverman, 1985). In the second short-range condition, the contrast of one set of patches was doubled (no spatial separation). Finally, in a second experiment in another group of infants we used long-range stimuli that were a factor of two higher in contrast.

**Figure 6:**
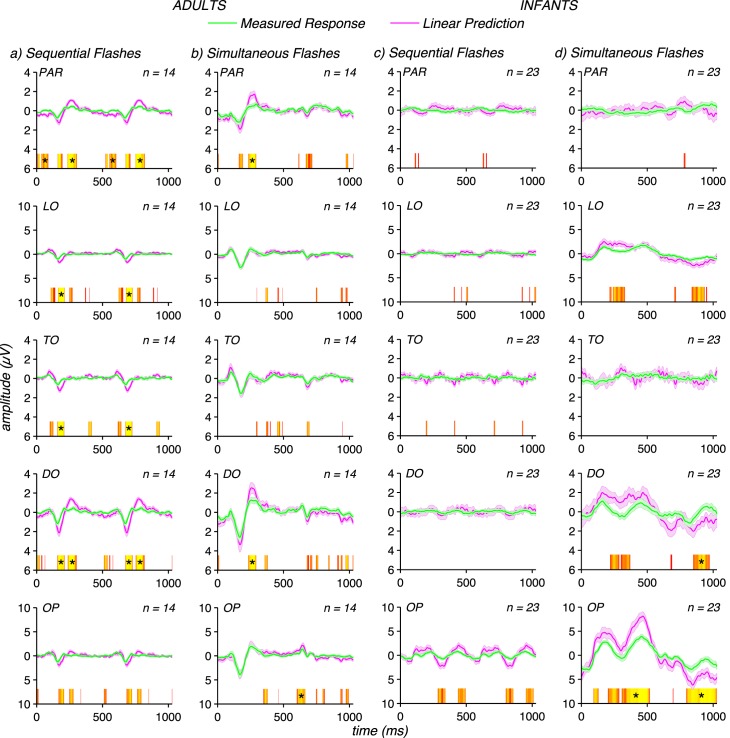
Measured responses (green curves) and their corresponding linear predictions (magenta curves) for adults (left) and infants (right) for short-range sequential flashes (panels a and c) and for short-range simultaneous flashes (panels b and d). The adult responses show similar magnitude subadditivity for short-range flashes as were seen for long-range flashes in Figure 3. Infants, by contrast show subadditivity of comparable magnitude to that seen in the adults, unlike what was observed under long-range conditions (Figure 3). Waveforms are group means for each group (infants and adults) over the five electrode ROIs described in the legend for Figure 3, with error bars representing the standard error of the mean. Significance of the differences between scaled responses and linear predictions are plotted above the abscissa, following the logic described for Figure 3.

Figure 6 shows data from the sequential and simultaneous short-range flashes (green curves), along with their corresponding linear predictions (magenta curves). Data from the adults are shown on the left (panels a and b) and the corresponding infant data are shown on the right (panels c and d). The adult pattern of subadditivity under the short-range condition is similar to that seen under the long-range condition (compare Figure 6a to Figure 3a for sequential flashes and Figure 6b to Figure 3c for simultaneous flashes). In contrast to the data collected under the larger separation used in the experiment shown in Figure 3, the infant short-range responses show stronger subadditivity under both sequential and simultaneous flash conditions (Figure 6c and d, respectively). In the long-range sequential flash condition (cf. Figure 3), subadditivity was not present at any time point for OP where the infant response is largest, but there were many points of significant difference in the short-range condition shown in Figure 6b, although these differences were not sufficiently large to survive run correction.

**Figure 7:**
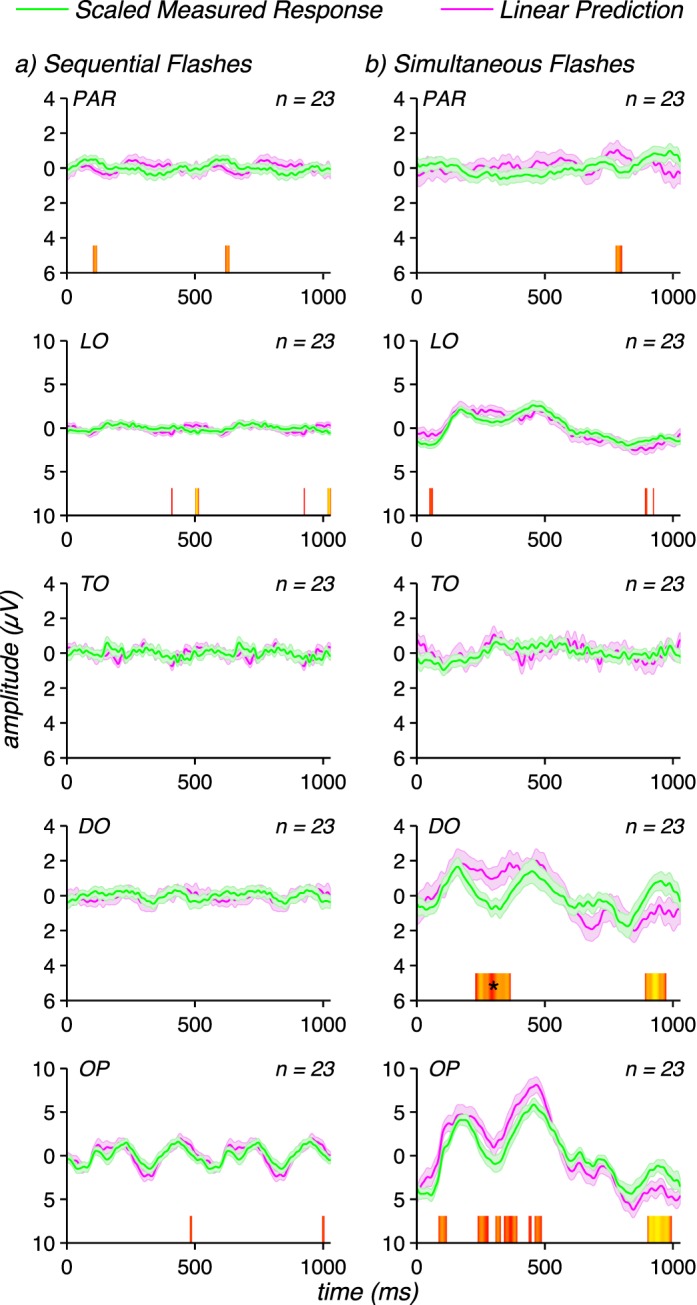
Scaled short-range flash responses from infants. Scaling the sequential flash responses by a factor of 2 (magenta curves) renders them similar to the corresponding linear prediction (green curves). Scaling the simultaneous flash responses by a factor of 1.5 renders them similar to the corresponding linear predictions. Waveforms are group means for each infant data set over the five electrode ROIs described in the legend for Figure 3, with error bars representing the standard error of the mean. Significance of the differences between scaled responses and linear predictions are plotted above the abscissa, following the logic described for Figure 3.

In the simultaneous flash condition, subadditivity was present after run correction for the short-range condition (Figure 6d), but not for the long-range condition (Figure 3d). Application of the adult factor of 2 scaling to the infant sequential flash data was sufficient to equate measured and predicted responses (see Figure 7, left). Applying the factor of 1.5 scaling from the adults to the infant simultaneous flash responses reduces the differences, but a substantial number of time points remain significant at the uncorrected *p* < 0.05. A larger scale factor would be necessary to completely eliminate the differences, indicating that in this condition the nonlinearity is, if anything, stronger in infants than adults. From this we conclude that the lack of subadditivity at 3 wavelengths in the infants (Figure 3b and d) is not due to a lack of contrast sensitivity or a general lack of saturation in the contrast response function. Spatially local suppressive interactions appear to be adult-like.

We then asked whether long-range interactions could be demonstrated with higher contrast stimuli, under the assumption that the efficacy of long-range connections may be weaker in infants compared to adults. This hypothesis was motivated by a computational model of long-range spatial interaction that was developed for collinear integration of Gabor patches separated by distances similar to our 3 lambda separation (Chen, Kasamatsu, Polat, & Norcia, 2001). In that model, responses to stimuli within the classical receptive field are subject to contrast normalization from a local normalization pool and are also modulated by long-range inputs. Here we reasoned that the long-range modulatory inputs might be weaker in infants and that their strength could be increased by increasing the stimulus contrast.

**Figure 8:**
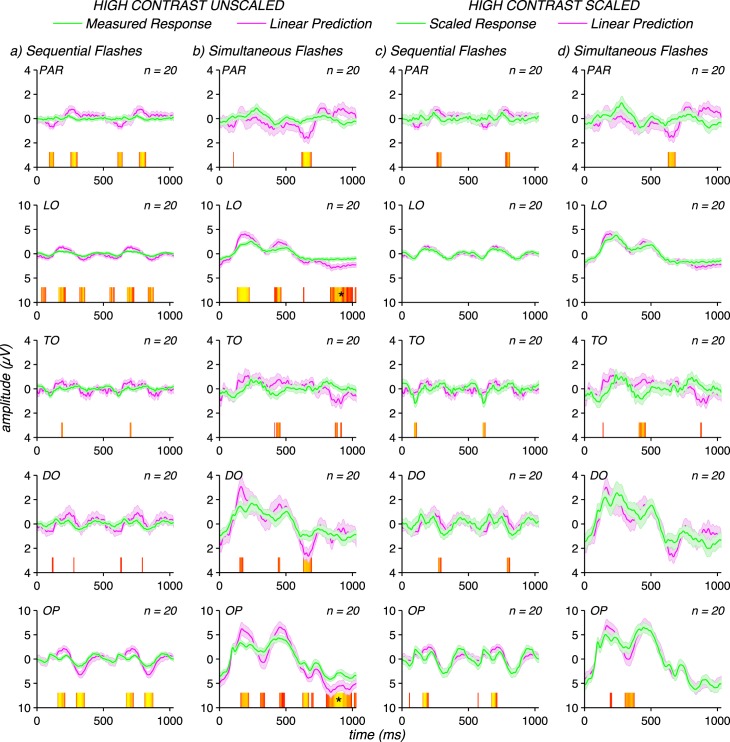
Infant evoked responses (green curves) measured at 80% contrast compared to corresponding linear predictions (magenta curves). Left panels show that the unscaled VEPs for sequential and simultaneous flash conditions are subadditive. Right panels show that the measured VEP can be equated to the linear prediction by scaling the sequential flash response by a factor of 2 and the simultaneous flash responses by a factor of 1.5. Waveforms are group means for each infant data set over the five electrode ROIs described in the legend for Figure 3, with error bars representing the standard error of the mean. Significance of the differences between scaled responses and linear predictions are plotted above the abscissa, following the logic described for Figure 3.

Figure 8 shows data from the sequential and simultaneous flash conditions for a group of 20 infants recorded at 80% rather than 40% contrast. Doubling to contrast of the stimuli led to infant responses that are subadditive for both sequential left and simultaneous flash conditions (see two left panels). This pattern contrasts with the data from the long-range, low contrast patch conditions in Figure 3 where there were no time points in OP or DO electrode ROIs that failed the additivity test. At high contrast, there are multiple failures of additivity, including points that survive run-correction for the simultaneous flash condition.

Application of the adult factor of 2 scaling for sequential and factor of 1.5 for simultaneous flashes to the high contrast infant data almost equalizes the linear prediction and measured responses. The direction of the prediction error is such that an even stronger nonlinear correction would be needed for the infants. These results thus suggest that infants have the necessary connectivity needed to generate long-range spatial temporal interactions, once stimulus contrast is high enough. Combining the results of the lower contrast short-range and higher-contrast long-range conditions, we conclude that infants have a specific immaturity in long-range spatiotemporal interactions that is independent of a simple reduction in their contrast sensitivity or a possible lack of saturation in the contrast response function.

## Discussion

Our results suggest that the relative maturity of spatiotemporal interactions in 4–7-month-old infants depends on spatial separation, with interactions between stimuli separated by small separations being mature and those between stimuli separated by longer distances being selectively immature. We chose to compare spatiotemporal interactions between sequentially presented stimuli as a probe of possible substrates for long-range motion processes that are known to exist in the adult. While we cannot ascribe the interactions observed here in either infant or adults to motion perception per se, the data place a bound on the availability of possible neural substrates for this type of perceptual processing. We find that stimuli with spatial configurations that are optimal for stimulation motion energy or short-range motion mechanisms are processed in an adult-like fashion, but that stimulus configurations designed to be poor activators of short-range motion mechanisms are not. The latter configuration, by contrast, is a sufficient stimulus to tap longer-range processes that are likely to be necessary precursors of an adult long-range perceptual motion system. We find that longer-range spatiotemporal interactions can be demonstrated in infants with high-contrast, but not moderate contrast, stimuli. Spatiotemporal interactions for spatially overlapping stimuli are adult-like at moderate contrast, so the immaturity at the longer separations is not a simple consequence of reduced contrast sensitivity in the infants or a lack of saturation in the contrast response function.

### Neural mechanisms of short- and long-range spatiotemporal interactions

The neural substrates for short- and long-range spatiotemporal interactions are likely to be different and thus, could have different developmental sequences. In the case of our short-range conditions where the stimuli are either completely or largely overlapped spatially, both sets of flashes will fall within classical receptive fields. Under our long-range conditions this overlap within classical receptive fields is lessened substantially. For example, Gabor patches similar to the ones used here when presented at the same 3-wavelength spatial offset from the center of the classical receptive field fail to elicit spiking in V1 neurons of the anesthetized cat. Nonetheless, stimuli at this remove from the center of the classical receptive field are capable of modulating responses to stimuli within the classical receptive field (Kasamatsu, Polat, Pettet, & Norcia, 2001; Polat, Mizobe, Pettet, Kasamatsu, & Norcia, 1998). These nonlinear modulatory effects have been modeled as a combination of a local contrast gain process operating within the classical receptive field and longer-range inputs acting in a multiplicative fashion (Chen et al., 2001). Similar multiplicative modulatory effects could underlie the non-linear spatiotemporal interactions observed here.

Functional spatiotemporal interactions may be mediated anatomically by either lateral horizontal connections within a visual area or by feedback connections from higher order cortical areas (Gilbert & Sigman, 2007). The feed-forward connections underlying classical receptive field organization have been shown to develop early in animal models (Singer, 1995) with basic feature selectivity being established near or before birth. Relevant to the present study, robust direction selectivity, for example, can be demonstrated by 1–2 weeks of age in macaque for short-range motion stimuli (Chino et al., 1997). Direction-selective evoked responses for grating stimuli have also been demonstrated in human infants starting at 6–10 weeks of age (Birch et al., 2000; Braddick, Birtles, Wattam-Bell, & Atkinson, 2005; Wattam-Bell, 1991). Consistent with this, we find adult-like spatiotemporal interactions for spatially overlapping stimuli.

Much less is known about the developmental status of horizontal intrinsic or feedback connections that could mediate longer-range spatiotemporal interactions. Horizontal intrinsic connections, while present at early ages, undergo experience-dependent maturation in a variety of species (Kennedy & Burkhalter, 2004), including human (Burkhalter, Bernardo, & Charles, 1993). Our understanding of the developmental status of the second anatomical substrate for long-range spatiotemporal interactions—feedback connections—is also still in a rudimentary state. In humans, the emergence of feedback connections between V2 and V1 lags that of feed-forward connections (Burkhalter, 1993). Data from developing infant macaques suggest that rudimentary feedback connections between V2 and V1 while being present at birth are refined over the first 8 weeks (Baldwin, Kaskan, Zhang, Chino, & Kaas, 2012). Given the commonly used rule of thumb that a week of infant monkey life is equivalent to a month of human life (Boothe, Dobson, & Teller, 1985) these feedback connections would be present, but likely still developing in 4–7-month-old human infants. It is of course difficult to infer the functional status of feedback connections simply on their presence or absence.

At the functional level, one index of longer-range spatiotemporal interaction is surround suppression—a suppressive influence of stimuli presented outside the classical receptive field on the response to stimuli presented within it. Surround suppression in V1 of macaque has been reported to be adult-like throughout the first 4 months of life (Kiorpes & Movshon, 2004). Another study (Zhang et al., 2005), however, found that surround strength increased over this period in V1 and that development of surround suppression in V2 was delayed relative to that present in V1. By contrast, V2 RF subfield structure was adult-like within the RF center. These surround-specific delays in development in higher-order visual areas could contribute to the relative differences we observed between short- and long-range spatiotemporal interaction.

Single-unit correlates of long-range apparent motion, as we have defined it, have not been found. An early extracellular recording study in striate cortex of the anesthetized cat (Ganz & Felder, 1984) presented flashing bars either singly or in temporal sequences. They found responses were subadditive when the sequence of flashes was in the nonpreferred direction of the cell. Although the flashes were widely separated (∼3°), they were nonetheless within the classical receptive field. Later work with apparent motion stimuli suggested that direction selectivity occurred over large spatial separations in macaque area MT where receptive fields are much larger than those in area V1 (Mikami, Newsome, & Wurtz, 1986a, 1986b). However, more recent work in macaque using stimuli that were more directly comparable in the two areas found that direction selectivity in V1 and MT/MST occurs only over a small and comparable range of temporal (less than about 100 ms) and spatial (less than about 1°) offsets (Churchland, Huang, & Lisberger, 2007; M. M. Churchland, Priebe, & Lisberger, 2005; Livingstone, Pack, & Born, 2001; Pack et al., 2006). It should be noted, however, that the more recent studies each used dense displays of small elements (random-dot fields or spatiotemporal white noise), unlike Mikami and colleagues (1986a, 1986b), who used single bars. In these more recent studies, the maximal spatial separation supporting direction selectivity in MT was clearly much smaller than the receptive field size. In addition, direction-selectivity in MT was found to be reversed in direction for opposite contrast stimuli, a property of cells in V1 (Born & Bradley, 2005; Livingstone & Conway, 2003; Livingstone et al., 2001) and of the motion energy model (Adelson & Bergen, 1985). Here we used stimuli that, while being a poor match to the properties of classical direction-selective receptive field mechanisms in early visual cortex, nonetheless give rise to a robust percept of apparent motion.

Other work in cat (Jancke et al., 2004; Rekauzke et al., 2016) and ferret (Ahmed et al., 2008) using voltage-sensitive dye imaging (VSDI) has suggested a second, qualitatively different type of direction selectivity may be represented in the spatiotemporal pattern of activity in large populations. Notably, for the present results, the measured VSDI response was approximately a factor of two smaller than the linear prediction based on responses to the flashes comprising the motion sequences presented singly. The magnitude of nonlinear suppression of the VSDI signal was thus similar to the one we find in the surface VEP.

The VSDI results have been modeled in several different ways: in the case of sequential-flash long-range apparent motion (Ahmed et al., 2008), they were modeled as being due to a moving peak of population activity in a recurrent feed-forward/feedback network of cells that are neither orientation- or direction-tuned (Deco & Roland, 2010). The network simulation coupled recurrent activity between two cortical areas, with the higher area having larger receptive fields than the lower area (Deco & Roland, 2010). In both higher and lower areas, apparent motion is represented by a spatial shift in the peak of the population response. Both feedback and relative delays between the two areas were necessary to simulate the pattern of VSDI results observed by Ahmed et al. (2008). In the case of the line-motion illusion, which also elicits a moving wavefront, the motion of the population-activity wavefront has been modeled as being due to recursive intra-areal horizontal connections, rather than between-areas feedback connections (Erlhagen & Jancke, 2004; Markounikau, Igel, Grinvald, & Jancke, 2010; Rangan, Cai, & McLaughlin, 2005). In each of these computational models, the underlying neurons are not themselves direction selective. As noted above, developmental immaturities in either the horizontal intrinsic or feedback connections critical to these models may explain the relative immaturity we have observed.

### The role of attention in apparent motion

As noted in the Introduction, it has been suggested on the basis of psychophysical studies with perceptually ambiguous stimuli that attention is a key component of the perceptions of long-range motion. Our results suggest that while attention does affect the response to long-range apparent motion stimuli (see Figure 5), it does not disproportionately contribute to the response as predicted by attention-based motion models. Notably, the present results were obtained with conventional apparent motion stimuli and not stimuli that were specifically designed to isolate attention-based motion mechanisms. Thus, while attention-based mechanisms are likely to contribute to the processing of our stimuli, they do not provide the sole, or even dominant, signal. Attention effects were in fact larger for stationary flashed stimuli than for sequential/moving stimuli.

Perhaps the strongest evidence for the relative independence of motion/sequence-related processing and attention in long-range apparent motion displays comes from the VSDI studies mentioned above in which the experimental results were obtained in anesthetized animals (Ahmed et al., 2008; Jancke et al., 2004). Robust traveling waves over the cortical surface consistent with the perceived direction of motion were observed. The fMRI data of Muckli et al. (2005) are also relevant to this question. They found that diverting attention away from apparent motion sequences or flashing control sequences reduced activation in both cases, but did not eliminate the larger activation for moving versus flickering stimuli. Here we find small, but measurable effects of attention that manifest as a gain-like change in the response amplitude. Attention has been previously shown to produce response-gain effects in the VEP with simple stimuli (Di Russo, Spinelli, & Morrone, 2001; Itthipuripat, Garcia, Rungratsameetaweemana, Sprague, & Serences, 2014; Lauritzen, Ales, & Wade, 2010). In our measurements, the underlying dynamics and sign of the spatiotemporal interaction are preserved independent of attention to the stimulus; only the relative magnitude is changed. Available computational models of the VSDI results (Deco & Roland, 2010; Markounikau et al., 2010) also suggest motion selectivity can be derived without the need for top-down inputs from attention, although such inputs can provide a modulatory influence. While feedback (either via top-down or lateral connections) appears to be important for long-range motion in models of long-range apparent motion, attention does not appear to be critical for the basic phenomenon, either theoretically or empirically.

## Conclusions

We have used additivity failures in VEP responses to grating patch arrays presented in isolation or in different spatiotemporal configurations to show that longer-range spatiotemporal interactions are subadditive. These nonlinear interactions are larger for sequential than simultaneous configurations, suggesting that they may contribute to motion processing. Infants demonstrate adult-like spatiotemporal interactions when spatial separation of stimulus elements is small. At larger spatial separations, adult-like interactions are only seen when stimuli have sufficiently high contrast. From these results, we conclude that longer-range spatiotemporal interactions are selectively immature in 4–7-month-old infants.
